# Processing, Characterization of *Furcraea foetida* (FF) Fiber and Investigation of Physical/Mechanical Properties of FF/Epoxy Composite

**DOI:** 10.3390/polym14071476

**Published:** 2022-04-06

**Authors:** Abhishek Sadananda Madival, Deepak Doreswamy, Srinivasulu Maddasani, Manjunath Shettar, Raviraj Shetty

**Affiliations:** 1 Department of Mechanical and Industrial Engineering, Manipal Institute of Technology, Manipal Academy of Higher Education, Manipal 576104, India; abhishek.madival@learner.manipal.edu (A.S.M.); manjunath.shettar@manipal.edu (M.S.); rr.shetty@manipal.edu (R.S.); 2 Department of Mechatronics, Manipal Institute of Technology, Manipal Academy of Higher Education, Manipal 576104, India; 3 Department of Chemistry, Manipal Institute of Technology, Manipal Academy of Higher Education, Manipal 576104, India; s.maddasani@manipal.edu

**Keywords:** *Furcraea foetida* fiber, retting, FT-IR, SEM, EDS, natural composite, tensile strength

## Abstract

In recent days the rising concern over environmental pollution with excessive use of synthetic materials has led to various eco-friendly innovations. Due to the organic nature, abundance and higher strength, natural fibers are gaining a lot of interest among researchers and are also extensively used by various industries to produce ecological products. Natural fibers are widely used in the composite industry as an alternative to synthetic fibers for numerous applications and new sources of fiber are continuously being explored. In this study, a fiber extracted from *the Furcraea foetida* (FF) plant is characterized for its feasibility as a reinforcement to fabricate polymer composite. The results show that the fiber has a density of 0.903 ± 0.07 g/cm^3^, tensile strength (σ_t_) of 170.47 ± 24.71 MPa and the fiber is thermally stable up to 250 °C. The chemical functional groups and elements present in the FF fiber are evaluated by conducting Fourier transform infrared spectroscopy (FT-IR) and energy dispersive spectroscopy (EDS). The addition of FF fibers in epoxy reduced the density (13.44%) and hardness (10.9%) of the FF/Epoxy (FF/E) composite. However, the void content (V_c_ < 8%) and water absorption (WA: < 6%) rate increased in the composite. The FF/E composite with 30% volume of FF fibers showed maximum σ_t_ (32.14 ± 5.54 MPa) and flexural strength (σ_f_: 80.23 ± 11.3 MPa).

## 1. Introduction

The constant rise in environmental pollution due to the extensive use of synthetic non-biodegradable materials is one of the major concerns across the globe. Various governmental bodies are imposing regulations to control the consumption of non-biodegradable materials and promoting the use of green materials is raising the demand for eco-friendly and bio-renewable products/materials [1,2]. Natural composites are considered as promising eco-friendly materials and continuous studies are being conducted to utilize them as an alternative to synthetic materials in various applications [3,4]. Natural composites are fabricated using fibers that are extracted from natural resources such as plants, animals and minerals. Some of the popular sources of plant fibers are flax, jute, sisal, agave, hemp, abaca, coir and banana plants [5]. The morphological properties of the plant fibers greatly depend on the extraction process, maturity of the plant and location of fiber in the plant. They may be extracted from their stem, seed, leaf, root and fruit. These fibers are attractive since they are abundant, low cost and show good mechanical properties [6,7,8]. Generally, fibers are extracted by water, dew, chemical, enzymatic and mechanical retting methods, etc. [9]. The natural-fiber-reinforced composites are advantageous than the synthetic versions because they are lightweight, non-toxic, biodegradable and exhibit acceptable strength and stiffness but may fail to provide resistance to moisture absorption, better wettability, strength and thermal stability.

The applications of natural fiber composites are exponentially increasing in recent years as their utilization in various fields, viz., in the preparation of automotive bumpers, dashboard structures [10,11,12], ballistic armors [13], keel beam, rudder and spoiler of aircraft components, satellite launch equipment [14], scaffolds in tissue engineering, dental restorative systems [15,16], thermal insulation/soundproof/roofing/door/window panels and internal structures of buildings [17], packaging [18], surfboards, fishing rod, musical instrument components, artifacts and tableware, etc., [19]. Natural fibers such as rice straw [20], cocoa bean [21], agave [22], wood [23], bamboo [24], etc., are explored for 3D printing applications [25]. Additionally, the researchers are focused on developing low-cost materials by utilizing rice straw [26], rice husk [27], sugarcane bagasse [28], olive [29] and pineapple [30] leaves, peanut shell, coconut shell, coffee hull, and other agro wastes [31,32,33,34] for other technical applications.

The literature on natural composites revealed that the fiber reinforcements such as flax, hemp, sisal, kenaf, etc. in polymers, improved the mechanical [35,36,37,38,39,40], tribological [41,42,43] and thermal [44,45,46] properties of polymer materials. However, these improvements greatly depend on the fiber morphology, chemical composition, thermal stability, alignment, aspect ratio and chemical treatments of fibers [47,48,49]. The porous structure of the natural fiber makes them suitable material for thermal insulation applications [50,51]. Few natural fibers show mechanical properties comparable with that of glass fibers [52,53,54,55,56,57]. The thermomechanical properties of natural composites are being enhanced by developing hybrid composites to suit the requirements of various applications [58,59,60].

**Figure 1:**
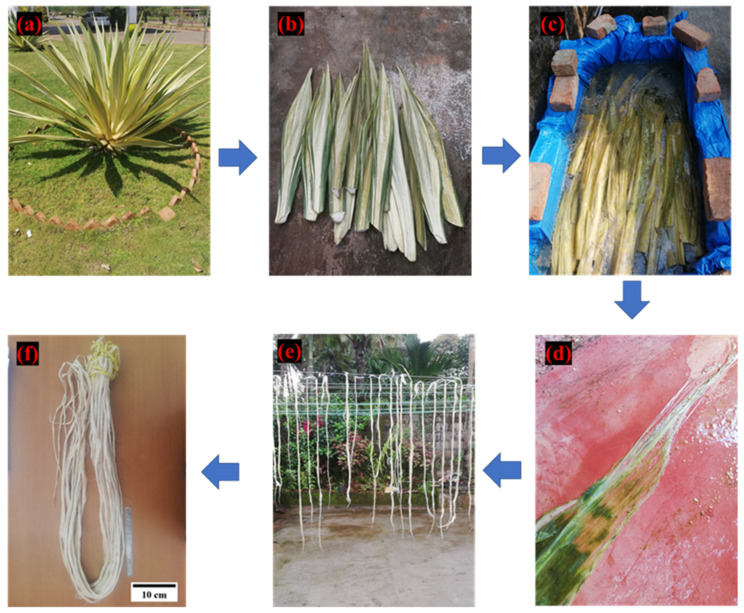
*Furcraea foetida* fiber extraction process (**a**) FF plant; (**b**) FF plant leaves; (**c**) water retting process; (**d**) separation of decayed substance; (**e**) drying of FF fiber; (**f**) extracted FF fiber bundles.

It is evident that studies are focused on developing sustainable and eco-friendly materials as an alternate to synthetic materials. Additionally, there is a quest for new sources of natural fibers which develop sustainable ecology and benefit society and the composite industry. The fibers derived from *Furcraea foetida* plant are unexplored for fabrication of polymer composites. The FF plant is a perennial subshrub plant that grows 4 to 6 feet tall (Figure 1a) and is widely seen in southern/western ghats of India. These plants are mainly used for fencing, to avoid soil erosion or landslides, and the root extracts are medically used to treat hepatitis, oedema, rheumatism, back pain and as a blood-purifying tonic [61,62,63]. The fibers extracted from the leaves of the FF plant are used for domestic applications to prepare twine cloth, mats and ropes, etc., [64]. The chemical composition of FF fibers is reported as it contains high cellulose content of 68.35 wt.%, hemicellulose of 11.46 wt.%, lignin of 11.46 wt.% and wax of 0.24 wt.%. The FF fibers exhibit good thermal stability ranging from 320 to 360 °C with an average surface roughness of 18.005 nm [65,66]. The high cellulose content (gives high strength) in FF fiber motivated us to select this as a reinforcing agent in epoxy composites. The preliminary study also showed excellent fiber properties. Therefore, the novel FF fiber as a polymer reinforcement is investigated for the first time by fabricating and evaluating the physio-mechanical properties of FF/E composites in the present study.

## 2. Materials

Epoxy resin (Lapox L12) and hardener (K6) (supplied by: Atul Pvt. Ltd., Gujrat, India) were used for the fabrication of test samples. The pot life of epoxy and hardener mixture is 30–40 min and the curing time is between 14 to 24 h at a temperature of 25 °C. The properties of epoxy and hardener are given in Table 1. The FF fiber is extracted from the plant leaves by the water retting process [67,68]. The matured FF plant leaves were chosen from the forests of Udupi region, Karnataka, India (Figure 1a). The leaves appear in a wedge shape with lengths varying from 90 to 170 cm and width 10 to 25 cm. The FF plant leaves (Figure 1b) were initially cleaned and then immersed in a water tank for five days to carry out the water retting process, as shown in Figure 1c. The surface layer of the leaves was decayed due to enzymatic actions during the water retting process which expose the inner fiber structure of the leaves [69]. The decayed greenish organic flesh of the leaves is separated from the fibers by gently crushing and brushing the leaf surface as shown in Figure 1d. The separated fibers are washed with distilled water to remove any organic deposits from the fiber surface. Finally, the extracted FF fibers are dried under sunlight for 24 h as shown in Figure 1e,f, which shows the extracted FF fiber bundles of length varying from 90 to 120 cm.

## 3. Materials Characterization

### 3.1. Fourier Transform-Infrared Spectroscopy

The chemical functional groups present in FF fiber are evaluated by conducting FT-IR. FF powder is mixed with KBr solution and the pellet is prepared using a simple press. The FTIR spectrum of FF fiber in the range of 4000 to 500 cm^−1^ wavenumber range of 32 scans with a resolution of 4 cm^−1^ is recorded using a spectrophotometer (IR Spirit, Shimadzu, Tokyo, Japan).

### 3.2. Thermogravimetric Analysis (TGA)

The TGA evaluates the rate of change in the mass of the material with respect to temperature and time in an inert atmosphere. The thermograms of the fibers and composite are recorded using a TGA apparatus (TGA 5500, TA Instruments, New Castle, DE, USA). A known weight of the fiber is taken in platinum crucible and is subjected to gradual heating at the rate of 10 °C/min up to 700 °C. Nitrogen is used as a purge gas with a flow rate of 20 mL/min to maintain the inert environment.

### 3.3. Scanning Electron Microscopy (SEM) and Energy Dispersive Spectroscopy

The surface morphology of the FF fiber is investigated by SEM (EVO MA18, Carl Zeiss Ltd., Cambridge, UK) at different magnification levels. The outer structure of the untreated FF fiber is examined at different fiber surface areas. The elements present on the surface of the FF fiber are evaluated by EDS using SEM and are measured in atomic and weight percentages.

## 4. Preparation of FF/E Composite

**Figure 2:**
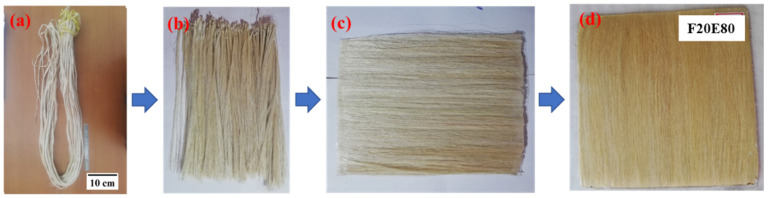
Process of FF fiber mat preparation (**a**) Extracted FF fiber bundles, (**b**) FF fiber strands, (**c**) FF fiber mat and (**d**) FF/E composite.

The unidirectional fiber mat is prepared by arranging the fiber strands on the table and fixing the ends of the fibers with adhesive tape [72] as shown in Figure 2a–c.

Further, the mold cavity is coated with PVA (polyvinyl alcohol)-releasing agent to easily remove the laminate after the hand-layup process [73,74,75]. The epoxy and hardener at the ratio of 1:10 are poured into a glass flask and the solution is mixed for 10 min to promote the chemical reaction. A thin layer of the epoxy mixture is applied on the mold surface and the FF fiber mat is then placed in the mold; using a brush, another layer of epoxy is coated on the fiber mat. This process is repeated until the desired thickness of the laminate is achieved. A hand roller and a soft brush are used to spread the epoxy on the fiber mat. Finally, a metal plate coated with releasing agent is placed on the mold to apply uniform pressure and remove the excess epoxy from the green laminate. The laminate is cured at room temperature for 24 h before removing it from the mold. Figure 2d shows the cured laminate and Table 2 shows the composition of test samples and their corresponding designations.

## 5. Density

FF fibers and FF/E test sample of known weight are immersed in a flask filled with distilled water and its volume of water displaced is recorded. The experimental density (ρ_e_) of these samples is calculated using Equation (1). The average density of test samples is calculated based on the readings of 10 trials. The theoretical density (ρ_t_) of the test samples is calculated by using the rule of mixture as given in Equation (2). The percentage of V_c_ in the prepared test samples is calculated by Equation (3), where ρ_m,f_ and V_m,f_ are the density (g/cm^3^) and volume fractions of matrix and fiber material, respectively [76,77].

(1) $$\rho_{e}{(\;g/\mathrm{cm}}^{3})\;=\;\mathrm{weight}\;\mathrm{of}\;\mathrm{the}\;\mathrm{sample}\;/\;\mathrm{volume}\;\mathrm{of}\;\mathrm{water}\;\mathrm{displaced}\;$$

(2) $$\rho_{t}{(g/\mathrm{cm}}^{3}{)=\rho }_{m}V_{m}{+\rho }_{f}V_{f}$$

(3) $$V_{c}=\frac{\rho_{t}-\rho_{e}}{\rho_{t}}\times \;100$$

## 6. Microhardness

The microhardness of the FF/E composite is determined by Vickers hardness (V_h_) tester (Make: MMT-X, Matsuzawa Co., Ltd., Toshima, Akita, Japan) as per ASTM E382-17 standard [78,79]. Indentation is produced on the test sample (50 mm × 25 mm × 3 mm) using a diamond indenter by applying a load of 100 g for a dwell period of 10 s. The indented region on the test sample surface is measured using the inbuilt microscope and the V_h_ is calculated using the Equation (4), where F_a_ and A_i_ are the applied force (N) and indentation area (mm^2^), respectively. The average V_h_ of tests samples is calculated by repeating the experimental trials five times on different indentation locations on the test sample.

(4) $$V_{h}=1.854\frac{F_{a}}{A_{i}^{2}}$$

## 7. Water Absorption Study

WA study of the test samples is conducted as per ASTM D570-98 standard using test samples of dimension 50 mm × 25 mm × 3 mm. Initially, test samples of known weight are soaked in tap water for 744 h. The moisture uptake in the test samples is recorded by measuring the weight of the test sample using electronic balance at a regular interval of 24 h. The WA rate of test samples is calculated by Equation (5) where W_1_ (g) and W_2_ (g) are the initial and final weight of the test samples [80,81].

(5) $$\mathrm{WA}\;(\%)=\frac{W_{2}{-W}_{1}}{W_{2}}\times \;100\;$$

## 8. Mechanical Properties

**Figure 3:**
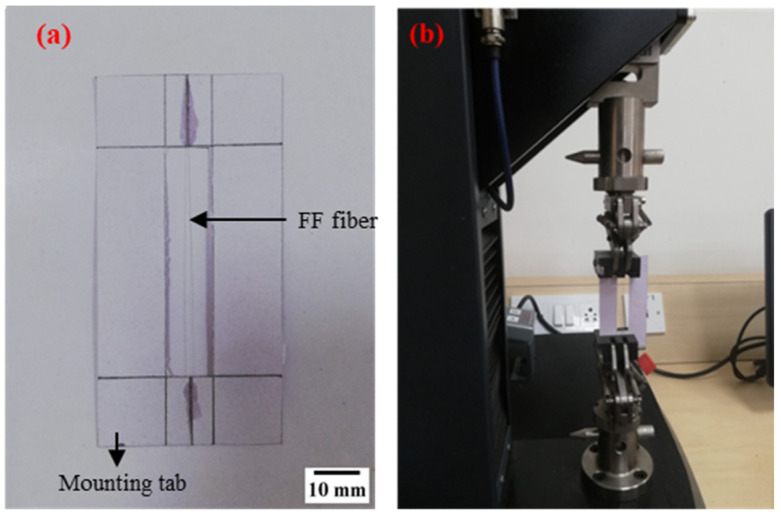
Tensile testing of fibers (**a**) FF fiber test sample and (**b**) Tensile testing machine.

The σ_t_ of the FF fiber is evaluated as per ASTM C1557-20 standard. A cardboard mounting tab is used to avoid the twisting or breaking of the fiber during the loading of fibers to the testing machine as shown in Figure 3a. The fiber is placed in the mounting tab by maintaining a gauge length of 50 mm and the ends of the fiber are constrained with epoxy adhesive. A 50 N capacity tensile testing machine (ETM-A, Shenzhen WANCE testing machine Co., Ltd., Beijing, China) is used to conduct the tensile test as shown in Figure 3b. The mounting tab is loaded to the testing machine and the sides of the mounting tab are cut to release the support of the mounting tab without damaging the fiber. The testing is conducted at a constant cross-head speed of 0.2 mm/min till the fiber breaks [82,83].

The σ_t_ and σ_f_ of the FF test samples are evaluated as per ASTM D 3039 and D 790 standards respectively. Universal testing machine (Make: UNITEK 9940, Fuel Instruments and Engineers Pvt. Ltd., Kolhapur, Maharastra, India) is used to conduct the test and a constant cross-head speed of 2 mm/min is maintained during the test till the test sample fail. Finally, the maximum failure load of test samples is recorded and the σ_t_ and σ_f_ of test samples are calculated using Equations (6) and (7), respectively. F_m_, A_c_, L, w and d are the maximum force (N), cross-sectional area (mm^2^), length (mm), width (mm) and thickness (mm), respectively. The theoretical tensile strength (σ_tt_) is calculated using Equation (8) where σ_r_,ε_r,_ V_r_ are max. stress, strain and V_f_ of reinforcement, respectively and E_m_ is the modulus of the matrix.

(6) $$\sigma_{t}{\;(N/\mathrm{mm}}^{2})=\frac{F_{m}}{A_{c}}$$

(7) $$\sigma_{f}{\;(N/\mathrm{mm}}^{2})=\frac{{3F}_{m}L}{{2\mathrm{wd}}^{2}}$$

(8) $$\sigma_{\mathrm{tt}}{=(\sigma }_{r}{)V}_{r}{+(\varepsilon }_{r}E_{m}{(1-V}_{r})\;$$

## 9. Results and Discussions

### 9.1. Fourier Transform Infrared Spectroscopy

**Figure 4:**
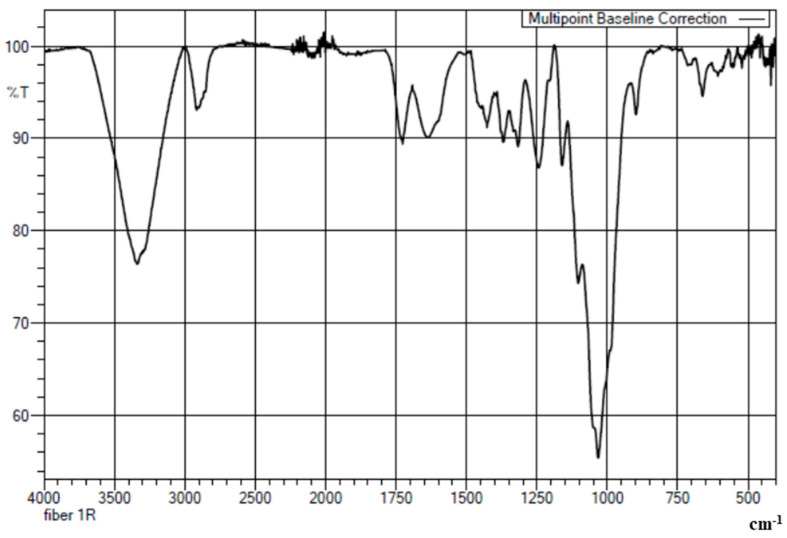
FTIR spectrum of FF fiber.

Figure 4 shows the FT-IR spectrum of FF fiber. The spectrum shows a strong relatively broad absorption peak at around 3335 cm^−1^, corresponding to the hydrogen-bonded O-H stretching. The peak at 2916 cm^−1^ is ascribed to the C-H stretching of the cellulose and hemicellulose molecules of the fiber. A peak observed at 1727 cm^−1^ is attributed to the –C=O moiety of the lignin present in the fiber. A peak at 1032 cm^−1^ is due to the –C-O- of the ether group and the bending vibrations of the –O-H group which are merged and appear as a strong band. Similar results were observed for fibers in the study conducted by tong et al., 2021 [84] (FF fiber), Ortega et al., 2019 [85] (Agave fiber) and Dizbay et al., 2018 [86] (Flax, Hemp, Sisal fiber).

### 9.2. Thermogravimetric Analysis

**Figure 5:**
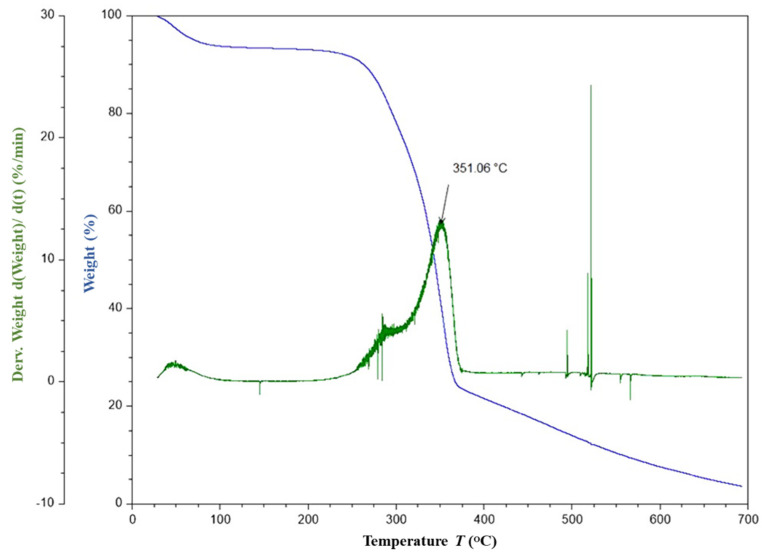
TG and DTG graph of FF fiber.

**Figure 6:**
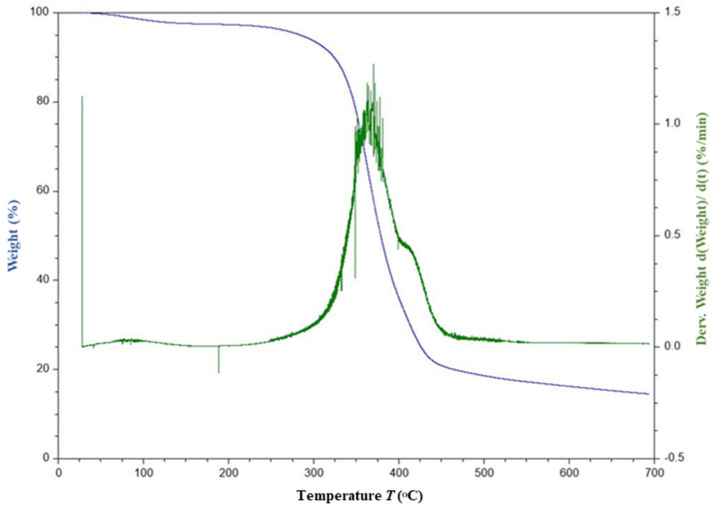
TG and DTG graph of F30E70 composite.

The TGA thermograms of FF fiber and F30E70 composite are given in Figure 5 and Figure 6, respectively. From the thermograms (Figure 5), the fibers are found to be thermally stable between 100 and 250 °C. The loss in mass up to 100 °C is about 8% is due to the evaporation of moisture. The onset temperature of the decomposition is observed at around 250 °C and the offset is about 375 °C. The decrease in mass between the temperature range of 250 to 375 °C involves two steps. In the first step, at about 278 °C, a mass loss of about 15% is observed, which may be due to the decomposition of glycosidic bonds present in the cellulose. In the second step, about 50% of major mass change is observed at 352 °C, which may be due to the depolymerization of hemicellulose and evaporation of α-cellulose moiety. The major mass change observed at 352 °C in the fiber is in concordance with the reported thermal degradation of cellulose I and α- cellulose [54,55] of other fibers. Similar final degradation temperatures are reported [87,88,89] in literature for different plant fibers such as Coccinia grandis stem (320 °C), bamboo (321 °C), hemp (308.2 °C), jute (298.2 °C) and kenaf (307.2 °C). In case of the FF/E composite, it is noticed that the onset of the degradation is about 250 °C and the offset is about 450 °C. The onset of degradation is unaltered in case of the F30E70 composite, but the offset temperature is increased by about 75 °C, which infers that the degradation of the composite taking place over a wide temperature range and may be due to breakage of the network of chemical bonds present in the epoxy thermoset. The maximum decrease in mass is observed at about 375 °C in the F30E70 composite, which is about 23 °C higher than the fiber. The results indicate that the FF fibers are suitable for reinforcement of composites and useful for high-temperature applications.

### 9.3. Energy-Dispersive X-ray Spectroscopy

**Figure 7:**
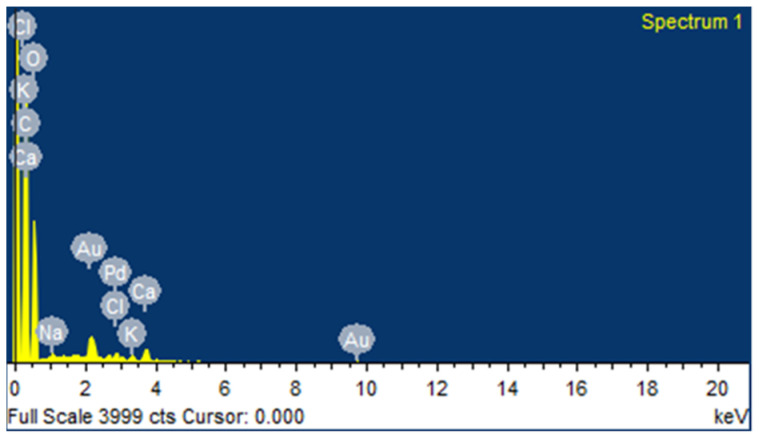
EDS spectrum of FF fiber.

Figure 7 shows the details of EDS analysis of FF fiber. The major compositional elements observed in FF fiber are C and O followed by Ca, Pd, K, Cl and Na. The weight % of C (46.02%) and O (41.93%) were observed to be slightly lower compared to the other reported natural fibers such as jute, cotton fiber, corn husk and hemp [90,91]. Table 3 shows the comparative atomic and weight percentage of different natural fibers. The traces of Au and Pd elements observed in the spectrum are due to the sputtering process, in which an ultra-thin coat of Au/Pd is applied to improve the conductivity of the test specimen during EDS.

### 9.4. Morphological Study of FF Fiber

**Figure 8:**
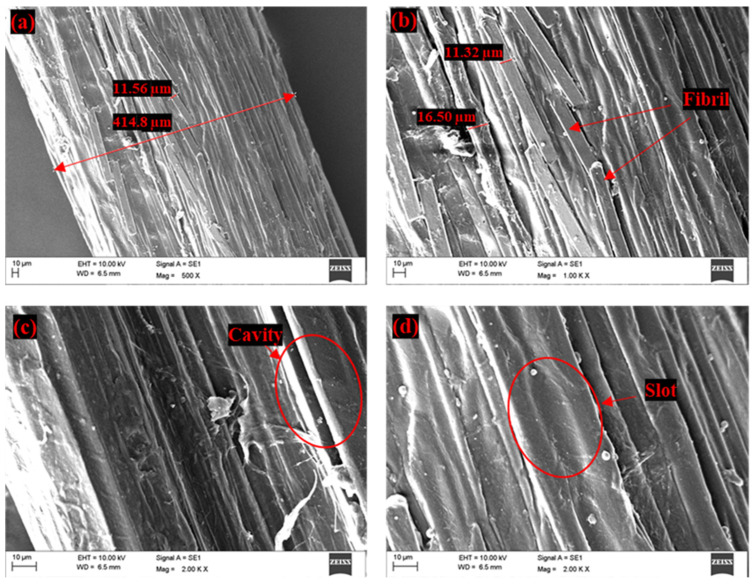
SEM images of untreated FF fiber surface (**a**) Diameter of fiber, (**b**) Diameter of fibrils, (**c**) Cavities observed in fiber and (**d**) Slots observed in fiber.

The Figure 8a–d shows the SEM images of FF fibers. As seen in Figure 8a, the FF fiber is observed to be made up of several closely packed fibrils (elementary fiber) which are bonded by pectin and other organic elements [92,93,94]. These fibrils are observed to be arranged with different length and diameter along the fiber direction.

**Figure 9:**
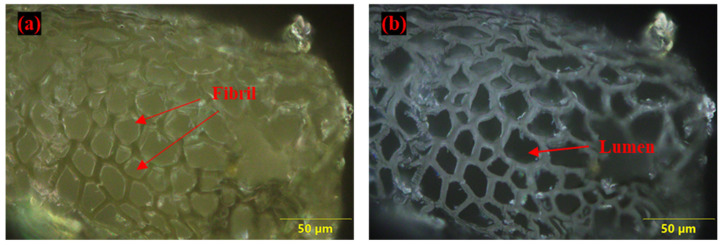
Microscopic images of cross-sectional area of FF fiber (**a**) Arrangement of fibrils in FF fiber bundle and (**b**) Lumens observed in fiber.

The diameter of the fibrils ranges from 5.83 to 19.9 µm as shown in Figure 8b. Whereas the diameter of FF fiber varies from 176.9 to 410.1 µm. The cavities and rectangular slots as observed in Figure 8c,d indicate the rough surface texture of FF fiber. This rough texture of FF fiber surface with cavities shows the better mechanical interlocking property of fiber with polymers. Similar observations on the surface morphology of FF fiber were made by Manimaran et al., 2018 [65] and Pathan et al., 2020 [66]. Figure 9a,b shows the microscopic images of cross-sectional area of the FF fiber. From Figure 9a it is seen that the fibrils appear to be of almost circular or filleted square shape. The closely packed fibrils are arranged in a honeycomb structure in the FF fiber bundle. The lumen structure observed in the Figure 9b is the intrinsic porosity of the FF fiber. Tong et al., 2021 [84] also observed similar lumen structure in FF fibers and the studies by Richely et al., 2021 [95], Madsen et al., 2013 [96], Hernandez et al., 2020 [97] confirm that the natural fibers have a porous structure.

### 9.5. Density

**Figure 10:**
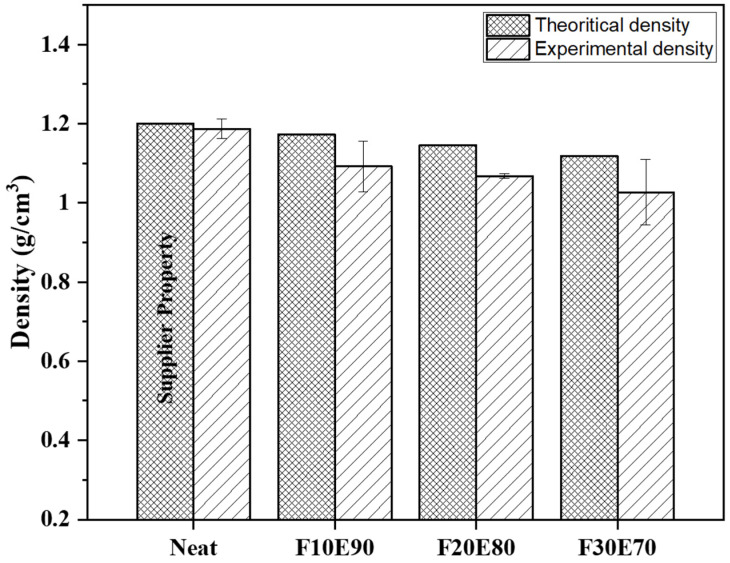
Density of test samples.

The Figure 10 shows the ρ_ct_ and ρ_ce_ of test samples. The average density of FF fiber is found to be 0.903 ± 0.07 g/cm^3^. However, the literature shows the variation of density of FF fiber from 0.778 to 0.891 g/cm^3^ [65,66].

**Figure 11:**
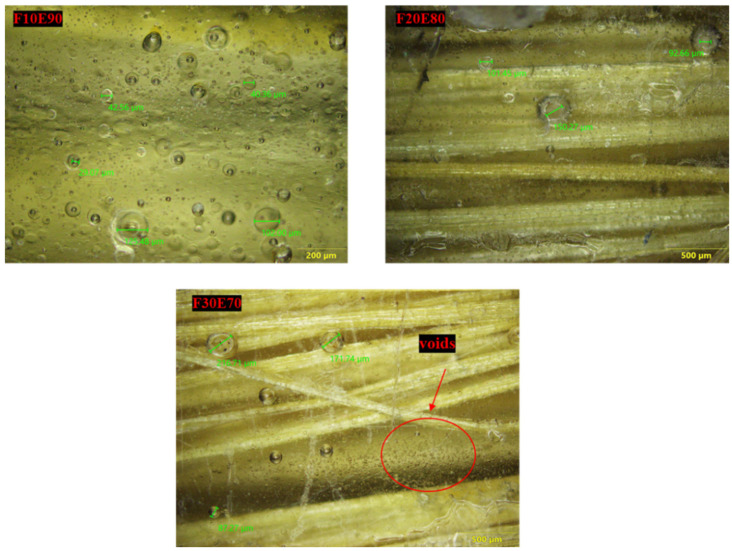
Microscopic images of test samples.

It is observed from the Figure 10 that the density of the test samples decreased with increase of FF fiber content. This reduction in ρ_ce_ is due to the lower density of FF fiber compared to epoxy material. The density of the F30E70 test sample is reduced by 13.44% compared to neat epoxy. The ρ_ce_ of the test samples is lower compared to ρ_ct_. This is mainly due to the formation of voids during the fabrication of test samples. The voids are developed due to the entrapment of gaseous volatiles which are released by the chemical reaction of epoxy and hardener during the fabrication. The FF fiber reinforcement acts as a barrier for the escape of gases and produces bubbles that form the voids in the test samples [36]. The percentage of void content in neat, F10E90, F20E80 and F30E70 is observed to be 1.06, 6.67, 6.69 and 7.47%, respectively. The size of the voids in the test samples is found to vary from 22.75 to 514.84 µm and appears globular in shape, as seen in Figure 11.

### 9.6. Water Absorption

**Figure 12:**
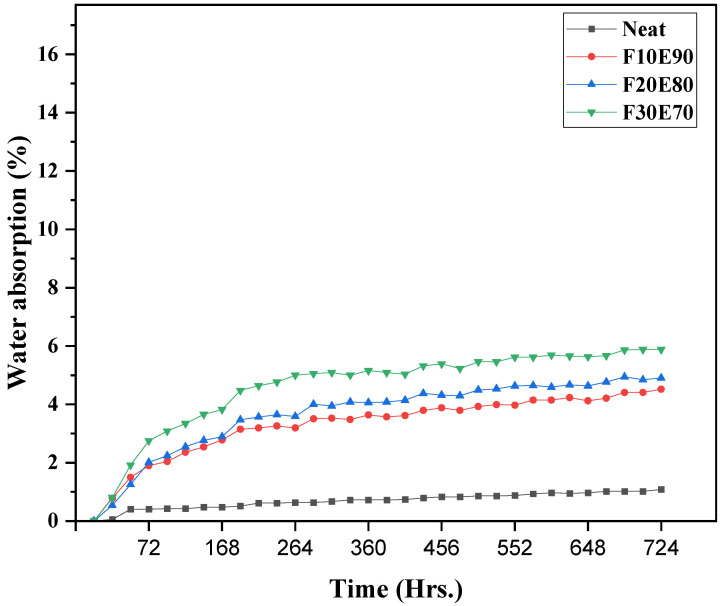
WA rate of test samples.

The water-resistant property of the test samples is studied by immersing the test samples in water at room temperature for 744 h. The Figure 12 shows the WA rate of test samples monitored at a regular interval of 24 h. It is observed that the WA rate of the test samples significantly increased up to 72 h of immersion period. After a certain period, the rate of absorption gradually dropped and remained almost constant. Beyond this peak level the moisture uptake in the test sample is almost negligible. It is observed from the figure that the addition of FF fiber in epoxy increases the WA rate of test samples. This is mainly due to the hydrophilic nature of FF fibers [98]. Additionally, the presence of voids, micro cracks and cavities between the fiber–matrix interphase influences the water absorption in test samples [99,100]. The WA rate in FF test samples is observed to be below 6%. Although the polymer materials are water resistant due to the voids, they showed a WA rate of 1.02% which is lowest among test samples. Whereas F10E90, F20E80 and F30E70 test samples showed WA rate of 3.75, 4.41 and 5.92%, respectively, with 31 days of immersion.

### 9.7. Microhardness

**Figure 13:**
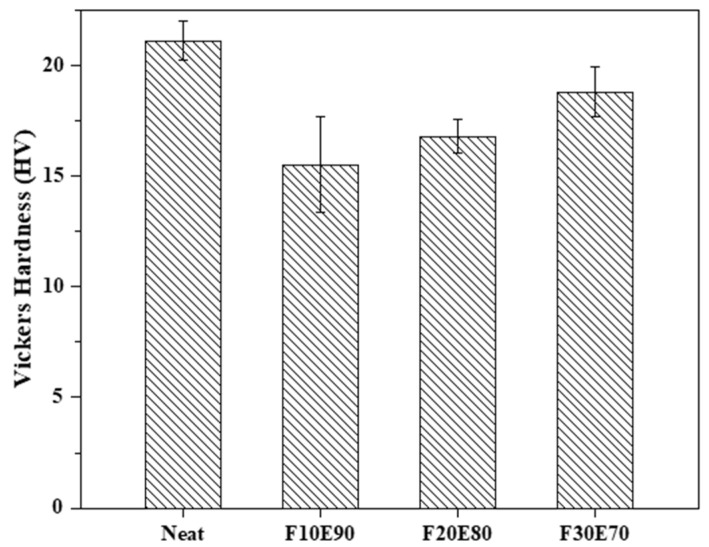
Hardness of test samples.

The Figure 13 shows the hardness of the test samples. The neat test sample showed the highest hardness of 21.1 ± 0.87 HV compared to other test samples. The neat epoxy laminate showed higher hardness (10.9%) compared to F30E70 composite. This reduction in hardness in FF/E composite is due to the higher amounts of micro-voids in the test samples. These micro-voids form weak surfaces and offer poor strength against penetration, thus reducing the hardness of the materials. However, the increase in FF reinforcement gradually improved the hardness of composites. At higher fiber content, the fibers are closely distributed and the interfacial adhesion of fibers improves and increases the composite’s hardness [101,102]. The hardness of F30E70 test sample is increased by 17.44% compared to the F10E90 test sample.

### 9.8. Tensile Strength of FF Fiber and FF/E Composite

**Figure 14:**
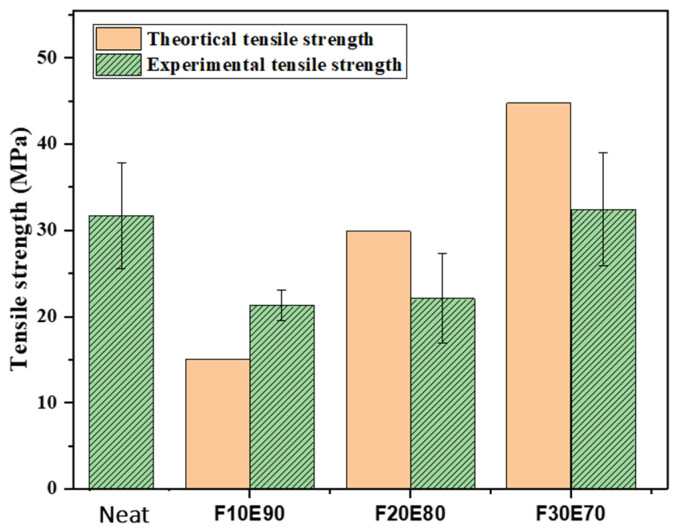
Theoretical and experimental tensile strength of test samples.

**Figure 15:**
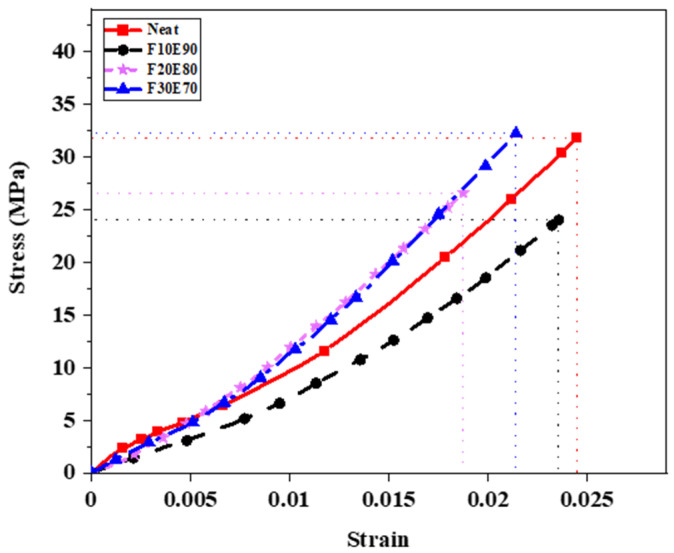
Stress–strain graph of test samples under tensile load.

The average σ_t_ of FF fiber with five experimental trials was found to be 170.47 ± 24.71 MPa. The Figure 14 shows the theoretical and experimental σ_t_ of the FF/E test samples. The F30E70 test sample showed maximum σ_t_ of 32.14 ± 5.54 MPa compared to neat, F10E90 and F20E80 composites. The interfacial region between the fiber and matrix produced weak interphase and reduced the strength of composites [103]. However, with the increase in fiber content the load applied was transferred to reinforced fiber and the strength of the composites improved [104]. The experimental σ_t_ of test samples was observed to be lower compared to σ_tt_ of test samples in most of the cases. This is due to voids and the heterogenous nature of FF fibers which affected the structural stability of the test samples [105]. The Figure 15 shows the stress–strain diagram of test samples subjected to tensile test. The neat epoxy test sample showed a maximum strain value of 0.025. It is observed that the addition of fibers decreased the strain. At highest fiber content of 30%, lowest strain is observed. The F30E70 test sample showed considerably higher strength compared to other test samples. The tensile properties of FF/E composite are comparable to different natural composites fabricated by Engin et al., 2019 [106], Cristiano et al., 2018 [107], Saba et al., 2019 [108], Mahesha et al., 2016 [72].

### 9.9. Flexural Strength of FF/E Composite

**Figure 16:**
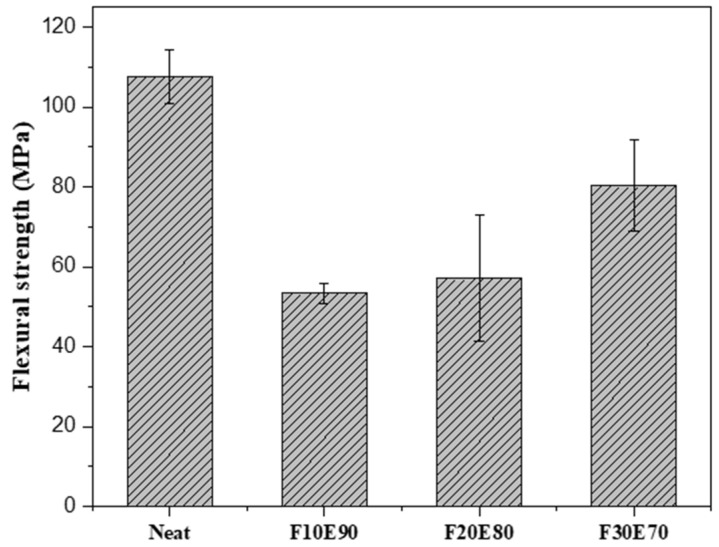
Flexural strength of test samples.

The Figure 16 shows the σ_f_ of FF/E test samples. The neat composite showed highest σ_f_ (107. 63 ± 6.69 MPa) compared to F10E90, F20E80 and F30E70 test samples. As explained in the earlier sections, the higher percentage of voids is responsible for the reducing the σ_f_ of FF/E composites compared to the neat test samples [109]. Although the FF/E test samples exhibited low σ_f_ compared to neat epoxy sample, the σ_f_ gradually improved with the increase in the FF fiber content. The addition of FF fiber volume from 10 to 30% improved the σ_f_ of the composite by 44.98%. The composite prepared by Jawaid et al., 2021 [60], Mohanavel et al., 2021 [110], Jumaidin et al., 2021 [111], Devireddy et al., 2021 [112] and Ajith et al., 2014 [113], showed comparable flexural strength to that of FF/E composites.

**Figure 17:**
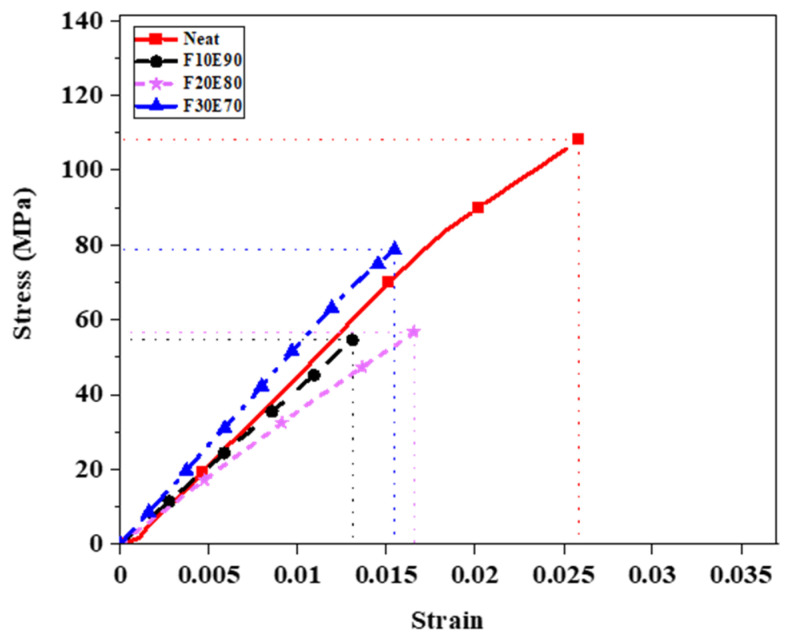
Stress–strain graph of test samples under flexural load.

The Figure 17 shows the stress–strain graph of test samples subjected to flexural load. The neat epoxy test samples showed maximum strain (0.0258). The strength of the FF/E composites is seen to be improved by the addition of FF fiber. The F30E70 test sample showed the highest strain of 0.0165 and strength of 78.45 MPa. It is observed from Figure 15 and Figure 17 that the tensile and flexural strength of F30E70 composite was higher compared to F10E90 and F20E80 composites. This is due to the higher fiber content and high crystalline nature of FF fibers [65], which are parallelly aligned in the composite.

### 9.10. Fractural Analysis of FF/E Composites

**Figure 18:**
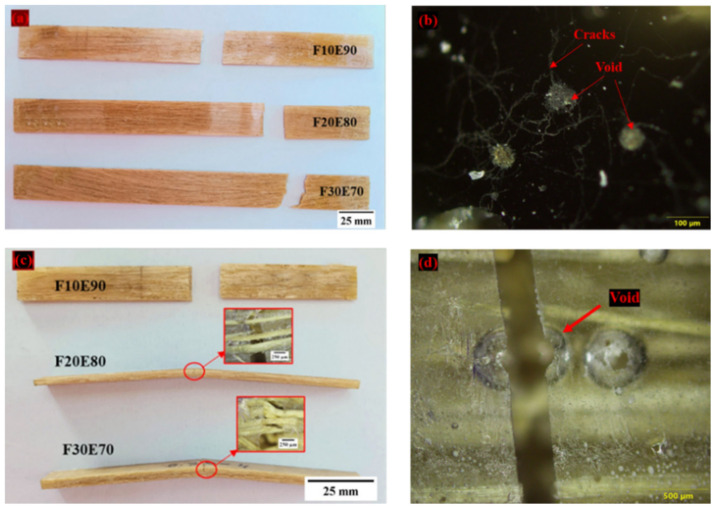
Fractured FF/E test samples subjected to (**a**,**b**) Tensile and (**c,d**) flexural load.

**Figure 19:**
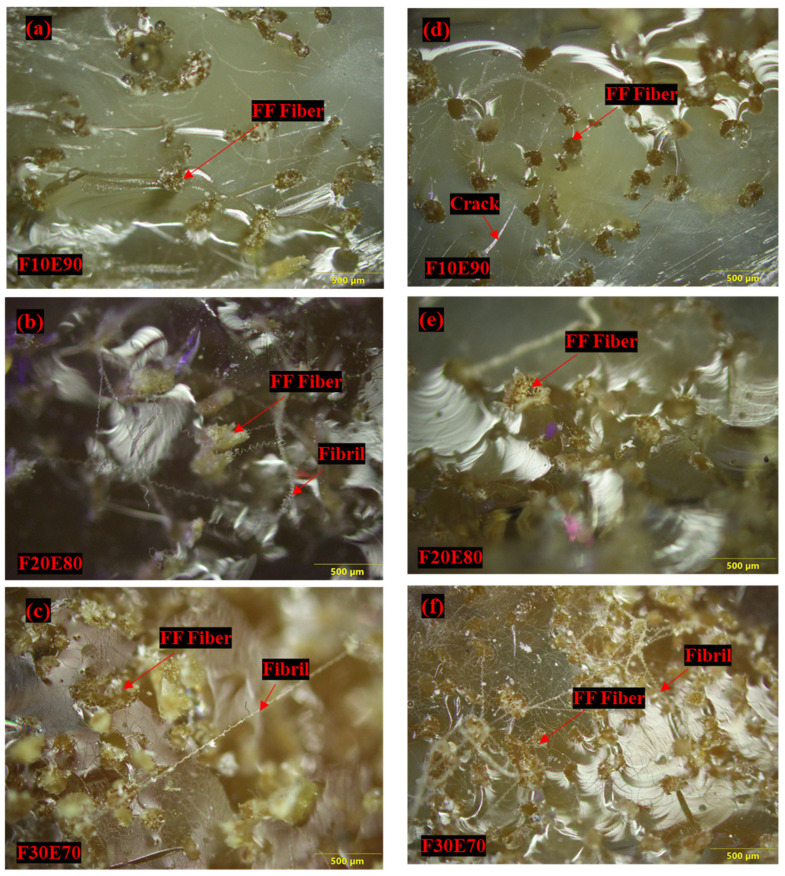
Microscopic images of FF/E composites subjected to (**a**–**c**) Tensile (**d**–**f**) Flexural load.

The Figure 18a,b shows the orthographic view of failed FF/E composite under tensile and flexural loads, respectively. The microscopic images of tensile test samples did not show delamination near the failure line, which indicates higher bond strength between fiber and matrix (Figure 18a). The Figure 18b shows that the failure line is horizontal and it appears to follow a weak interface located near the failure area. As seen from Figure 18c, the F10E90 test sample subjected to flexural load fractured at the center of the specimen. However, in F20E80 and F30E70 the test samples showed that the fiber phase was intact even after the failure of the composite. This show that the failure of test samples occurred due to the fracture of matrix phase. The micro voids present in the test sample acted as stress concentrators and the applied load was transmitted between these weak locations, which resulted in the fracturing of the matrix phase (Figure 18d) [114]. Figure 19a,b shows the optical microscope images of fractured surfaces subjected to tensile and flexural load, respectively. The fibers are observed to be intact and no signs of fiber pull-out are observed from the images. The fibrils observed on the surfaces of fractured surface areas indicate that the applied load is successfully transferred to the fiber phase [115].

## 10. Conclusions

The FF plants which grow abundantly in the forest regions are locally used for domestic purposes. The morphological observations such as high cellulose content, rough surface structure and thermal stability of FF fibers indicate good fiber properties. These FF fibers can be seen as a potentially inexpensive source of reinforcement for polymer composites. In this regard, the effectiveness of FF fibers in reinforcing epoxy material is evaluated by fabricating and testing the physical/mechanical properties of FF/E composites for the first time. This study aims to extract, characterize the FF fibers and evaluate the effect of FF fiber content on the physio-mechanical properties of FF/E composite. The observations made from the microscopic images of the tensile and flexural test failed samples show good bonding between FF fiber and epoxy. Additionally, the FF reinforcement considerably improved the mechanical properties of the epoxy material.

Further, improving the quality of FF fiber by chemical treatments is expected to enhance the properties of the composite. The prepared FF/E composite shows promising results and can be used to develop cost-effective materials for different eco-friendly applications. Based on the present investigation, the following conclusions are derived.

- The FF fiber extracted from its plant’s leaf using the water retting process showed a density of 0.903 ± 0.07 g/cm^3^, indicating its usability in lightweight applications. The higher amount of carbon and oxygen observed in EDS signifies the organic nature of the FF fibers.
- The rough surface texture with rectangular-shaped slots observed in the microscopic images of FF fiber indicates better interlocking property and higher compatibility as reinforcement material.
- The TG analysis showed that the FF fiber is thermally stable between the temperature range of 100 to 250 °C. and the maximum thermal degradation for FF fiber is observed at 352 °C.
- The addition of FF fibers decreased the density of FF/E composites up to 13.44% due to the lower density of FF fiber compared to epoxy and the presence of voids in the test samples. Additionally, these voids increased with FF fiber content and among them, F30E70 showed the highest void content (7.47%).
- The WA rate in the test samples increased with FF fiber concentration due to the hydrophilic nature of FF fiber. However, the maximum WA rate observed in the FF/E composite was <6%.
- The mechanical properties of FF/E composite gradually improved with FF fiber content. The F30E70 showed the highest σ_t_ (32.14 ± 5.54 MPa) and the neat epoxy showed the highest σ_f_ (107.63 ± 6.69 MPa) compared to other test samples. Additionally, the F30E70 composite showed slightly higher thermal stability than FF fibers.

## Figures and Tables

**Table 1:** Properties of epoxy [70,71].

Properties	Range
Density of epoxy (L-12) at 25 °C	1.1–1.2 g/cm^3^
Density of hardener (K-6) at 25 °C	0.95–1.1 g/cm^3^
Tensile strength	55–70 MPa
Flexural strength	120–140 MPa
Impact strength	17–20 KJ/m^2^
Thermal conductivity	0.211 kCal/m h °C
Coefficient of liner thermal expansion	64–68 10^−6^/°C
Water absorption (25 °C/24 h)	0.5 *w/w* % (Max)

**Table 2:** Coding of test samples.

Sl. No	Sample Code	FF Fiber (V_f_)	Epoxy (V_m_)
1	Neat	0	1
2	F10E90	0.10	0.90
3	F20E80	0.20	0.80
4	F30E70	0.30	0.70

**Table 3:** Weight and atomic percentages of FF and other natural fibers [65].

Element	*Furcraea Foetida*		Jute		Cotton	
	Weight (%)	Atomic (%)	Weight (%)	Atomic (%)	Weight (%)	Atomic (%)
C	46.02	58.03	55.68	62.72	46.1	53.2
O	41.93	39.69	43.89	37.11	53.9	46.8
Na	0.42	0.28	-	-	-	-
Cl	0.53	0.23	-	-	-	-
K	0.78	0.30	-	-	-	-
Ca	1.93	0.73	-	-	-	-

## Data Availability

Data sharing is not applicable.
